# Minding Averages: Comment on “Experimental Design and Power Calculation in Omics Circadian Rhythmicity Detection Using the Cosinor Model” by Zong et al.

**DOI:** 10.1002/sim.70044

**Published:** 2025-05-21

**Authors:** Pål O. Westermark

**Affiliations:** ^1^ Working Group Husbandry & Health Research Institute for Farm Animal Biology (FBN) Dummerstorf Germany; ^2^ Institute for Biostatistics and Informatics in Medicine and Ageing Research Rostock University Medical Center Rostock Germany

## 1 Introduction

We read with great interest the article by Zong et al. [1], where the authors explored the statistical power of the standard cosinor model for detection of biological rhythms with a focus on circadian rhythms and its implications for experimental design, that is, the choice of the circadian time or zeitgeber time (ZT) points at which measurements are made. As the authors rightfully point out, power analysis has been an overlooked topic in chronobiology and biological rhythms research. Except for being necessary for the planning and design of experiments, power analysis has the additional potential to explain, for example, inconsistencies and a certain lack of reproducibility in studies of circadian or diurnal rhythmicity in gene expression [2, 3]. However, we must conclude that the expression for the noncentrality parameter that is necessary to compute power has an incorrect form (part of Theorem 2 in their article), which affects all cases when the experimental design is non‐centered (see below for precise definition of centered design in the context of the cosinor model). Here, we present a correct derivation of the noncentrality parameter, which requires some extra steps. As we further show, the resulting discrepancy between the correct and incorrect forms in computed power can be large. However, for the data analyses presented by Zong et al. [1], we do not foresee large discrepancies, so that their results for non‐centered designs probably still hold qualitatively, although that would need to be numerically verified in a rigorous manner.

## 2 Background

For reference, what we here call the *standard cosinor model* assumes n measurements $y_{i}$ taken at times $t_{i}$, i=0,…,n−1, and

$$y_{i}=m+a_{1}\cos\omega t_{i}+a_{2}\sin\omega t_{i}+\epsilon_{i}$$

with angular frequency ω=2π/T, T being the period of the rhythm under investigation, and assuming independent zero‐centered random variation terms $\epsilon_{i}$ with constant variance $\sigma^{2}$, in standard cosinor analysis assumed to have a Gaussian distribution $N\left(0,\sigma^{2}\right)$. Measurements, time points, and random variation terms are collected in (column) vectors y, t, and ϵ, respectively, so that the statistical model is

y=Xβ+ϵ

with $\beta ={\left(m,a_{1},a_{2}\right)}^{T}$, and with the design matrix

$$X=\left(\begin{array}{ccc} 1 & \cos\omega t_{1} & \sin\omega t_{1}\\ \vdots & \vdots & \vdots \\ 1 & \cos\omega t_{n} & \sin\omega t_{n} \end{array}\right)=\left(X_{1}\;X_{2}\right)$$

The design matrix is here decomposed into $X_{1}={\left(1,\ldots ,1\right)}^{T},$ and $X_{2}$ formed by the cosine and sine terms at the measurement time points. We have $a_{1}\cos\omega t+a_{2}\sin\omega t=A\cos\left(\omega t-\phi \right)$, so that the cosinor model accommodates rhythms with any amplitude $A=\sqrt{a_{1}^{2}+a_{2}^{2}}$ and any phase ϕ with $\tan\;\phi =\frac{a_{2}}{a_{1}}$, and with a location parameter m. In standard cosinor analysis, the null hypothesis is $\beta ={\left(\beta_{1}\;\beta_{2}\right)}^{T}$ with mean $\beta_{1}=m$ and $\beta_{2}={\left(a_{1}\;a_{2}\right)}^{T}=0,$ the null model thus has only 1 free parameter. The alternative hypothesis is the full model with all 3 parameters free. Estimates of parameters, which for standard cosinor are least squares (LS) estimates, are denoted with hats, for example, $\hat{\beta }$. The unbiased LS estimates for the full model are expressed by the normal equation $\hat{\beta }={\left(X^{T}X\right)}^{-1}X^{T}y.$ For the null model, we in the same way have ${\hat{\beta }}_{1}={\left(X_{1}^{T}X_{1}\right)}^{-1}X_{1}^{T}y$. After finding estimates, hypothesis testing involves the F statistic

(1) $$F=\frac{\left(\mathrm{SS}\left({\hat{\beta }}_{1}\right)-\mathrm{SS}\left(\hat{\beta }\right)\right)/\left(p-q\right)}{\mathrm{SS}\left(\hat{\beta }\right)/\left(n-p\right)}$$

with p and q being the number of parameters in the full model and the null model, respectively; in standard cosinor analysis we have p=3 and q=1. Here, $\mathrm{SS}\left({\hat{\beta }}_{1}\right)={\left(y-X{\hat{\beta }}_{1}\right)}^{T}\left(y-X{\hat{\beta }}_{1}\right)$ is a sum of squares error (SSE) for the null model, $\mathrm{SS}\left(\hat{\beta }\right)$ is the SSE for the full model. Hypothesis testing against the null model proceeds by evaluating the resulting central F(p−q,n−p) distribution.

### 2.1 A Fallacy

To analyze and obtain expressions that describe statistical power, we need to consider any cases where $\beta_{2}\neq 0$. As outlined below, this leads to a noncentral F distribution, which for practical cosinor power analysis should be expressed as a function of $\beta_{2}$, which is readily related to amplitude and phase. The starting point is the reduction in sum of squares which, due to the geometry of least squares, can be decomposed by Pythagoras' theorem in ${\mathbb{R}}^{n}$: $\mathrm{SS}\left({\hat{\beta }}_{1}\right)-\mathrm{SS}\left(\hat{\beta }\right)={\left(X_{1}{\hat{\beta }}_{1}-X\hat{\beta }\right)}^{T}\left(X_{1}{\hat{\beta }}_{1}-X\hat{\beta }\right)$. Since $X\hat{\beta }=\left(X_{1}\;X_{2}\right)\left(\begin{array}{c} {\hat{\beta }}_{1}\\ {\hat{\beta }}_{2} \end{array}\right)$, it is now tempting to conclude that $\mathrm{SS}\left({\hat{\beta }}_{1}\right)-\mathrm{SS}\left(\hat{\beta }\right)\overset{\text{fallacy}}{=}{\hat{\beta }}_{2}^{T}X_{2}^{T}X_{2}{\hat{\beta }}_{2}$. This leads to the incorrect noncentrality parameter of Lemma 3 of Zong et al. [1], in turn leading to their invalid Theorem 2. The error stems from the fact that the estimate ${\hat{\beta }}_{1}$ is in general not the same for the null and full models: we denote them ${\hat{\beta }}_{1,\text{null}}$ and ${\hat{\beta }}_{1,\text{full}}$. The intuition for the reason is that the sine and cosine terms do not necessarily sum up to zero, and may thus contribute to the mean of the $\hat{y}$ estimates, so that ${\hat{\beta }}_{1,\text{full}}$ becomes different from ${\hat{\beta }}_{1,\text{null}}$.

### 2.2 A General Procedure Based on Orthogonalization

Procedures for finding the correct expression for the reduction in sum of squares as a function of $\beta_{2}$ are described in textbooks [4]. For the case of the standard cosinor model, this amounts to a reparameterization resulting in a straightforward centering of $X_{2}$, but for future reference, we proceed in a quite general manner and allow any $X_{1}$, which could describe trends, blocking, or other nuisances. The general reparameterization is in some fields known in the context of the Frisch‐Waugh‐Lovell theorem. The main idea is to reparameterize by subtracting the projection of $X_{2}$ on $X_{1}$ from $X_{2}$, to create a modified design matrix $\left(X_{1}\;Z_{2}\right)$ for the full model. In this way, $X_{1}$ and $Z_{2}$ are orthogonal, $X_{1}^{T}Z_{2}=0$. We shall see that $\mathrm{SS}\left({\hat{\beta }}_{1}\right)-\mathrm{SS}\left(\hat{\beta }\right)={\hat{\beta }}_{2}^{T}Z_{2}^{T}Z_{2}{\hat{\beta }}_{2}$, which leads to a correct expression for the noncentrality parameter necessary for power analysis. To show this, we introduce a change of basis transition matrix

$$U≔\left(\begin{array}{ll} I & -{\left(X_{1}^{T}X_{1}\right)}^{-1}X_{1}^{T}X_{2}\\ 0 & I \end{array}\right)$$

This reparameterization puts $\mathrm{XU}=\left(X_{1}\;Z_{2}\right)$, with

(2) $$Z_{2}=\left(I-X_{1}{\left(X_{1}^{T}X_{1}\right)}^{-1}X_{1}^{T}\right)X_{2}$$

We then have the equivalent reparameterized model $X\beta =\left(\begin{array}{ll} X_{1} & Z_{2} \end{array}\right)U^{-1}\beta$, with new parameters

$$U^{-1}\beta ≔\left(\begin{array}{l} \lambda \\ \beta_{2} \end{array}\right)=\left(\begin{array}{l} \beta_{1}+{\left(X_{1}^{T}X_{1}\right)}^{-1}X_{1}^{T}X_{2}\beta_{2}\\ \beta_{2} \end{array}\right)$$

Importantly, $\beta_{2}$ and thus the estimates of amplitude and phase are the same in both parameterizations. We have ${\left(\begin{array}{ll} X_{1} & Z_{2} \end{array}\right)}^{T}\left(\begin{array}{ll} X_{1} & Z_{2} \end{array}\right)≔D$, where D is block‐diagonal because of the introduced orthogonality $X_{1}^{T}Z_{2}=0$. With this in mind, the parameter estimates for the reparameterized model follow immediately from the LS normal equations:

$$\left(\begin{array}{c} \hat{\lambda }\\ {\hat{\beta }}_{2} \end{array}\right)=\left(\begin{array}{cc} {\left(X_{1}^{T}X_{1}\right)}^{-1} & 0\\ 0 & {\left(Z_{2}^{T}Z_{2}\right)}^{-1} \end{array}\right)\left(\begin{array}{c} X_{1}^{T}\\ Z_{2}^{T} \end{array}\right)y$$

This shows that the estimates of λ stay the same for any $X_{2}$, a consequence of the introduced orthogonality and block‐diagonal form of D. Since the reparameterization does not affect the column span of the design matrix, sum squared errors for the null and full models are independent of reparameterization, and

$$\mathrm{SS}\left({\hat{\beta }}_{1}\right)-\mathrm{SS}\left(\hat{\beta }\right)={\left(X_{1}\hat{\lambda }-X\left(\begin{array}{l} \hat{\lambda }\\ {\hat{\beta }}_{2} \end{array}\right)\right)}^{T}\left(X_{1}\hat{\lambda }-X\left(\begin{array}{l} \hat{\lambda }\\ {\hat{\beta }}_{2} \end{array}\right)\right)={\hat{\beta }}_{2}^{T}Z_{2}^{T}Z_{2}{\hat{\beta }}_{2}$$

Observing that ${\left(X^{T}X\right)}^{-1}=UD^{-1}U^{T},$ we obtain

$${\hat{\beta }}_{1,\text{full}}={\hat{\beta }}_{1,\text{null}}-{\left(X_{1}^{T}X_{1}\right)}^{-1}X_{1}^{T}X_{2}{\hat{\beta }}_{2}$$

implying that the noncentrality parameter of Lemma 3 and Theorem 2 of Zong et al. [1] is incorrect unless $X_{1}^{T}X_{2}=0$, which is the defining case for orthogonal designs. For the standard cosinor model, we have

$${\hat{\beta }}_{1,\text{full}}={\hat{\beta }}_{1,\text{null}}-{\hat{a}}_{1}\mu_{\cos}-{\hat{a}}_{2}\mu_{\sin}$$

where we have defined $\mu_{\cos}=\frac{1}{n}\sum_{i}\cos\omega t_{i}$, and $\mu_{\sin}=\frac{1}{n}\sum_{i}\sin\omega t_{i}$, the averages of the model's rhythmic components taken at the measurement time points. If $\mu_{\cos}=\mu_{\sin}=0$, we say that the design is centered, and only in that case, ${\hat{\beta }}_{1,\text{full}}={\hat{\beta }}_{1,\text{null}}$ and the noncentrality parameter in Theorem 2 of Zong et al. [1] is correct.

We finally observe that by standard theory for probability and linear models, ${\hat{\beta }}_{2}∼N\left(\beta_{2},\sigma^{2}{\left(Z_{2}^{T}Z_{2}\right)}^{-1}\right)$. Consequently, ${\hat{\beta }}_{2}^{T}Z_{2}^{T}Z_{2}{\hat{\beta }}_{2}/\sigma^{2}∼\chi^{2}\left(p-q,\delta^{2}\right)$, a noncentral $\chi^{2}$ distribution with p−q degrees of freedom and noncentrality parameter

(3) $$\delta^{2}=\beta_{2}^{T}Z_{2}^{T}Z_{2}\beta_{2}/\sigma^{2}$$

For power analysis, one considers a given effect size (values of $\beta_{2}$ and σ) and observes that Equation (1) leads to an $F\left(p-q,n-p,\delta^{2}\right)$ distribution. A special case of this general exposition is hypothesis testing against the null model, where $\beta_{2}=0$, which leads to the central F distribution, $\delta^{2}=0$.

### 2.3 Correct Power Expression for the Standard Cosinor Model

For the standard cosinor model, results are straightforward and amount to a centering of the rhythmic terms, leading to a variance–covariance matrix. Equation (2) leads to

$$Z_{2}=\left(\begin{array}{cc} \cos\omega t_{1}-\mu_{\cos} & \sin\omega t_{1}-\mu_{\sin}\\ \vdots & \vdots \\ \cos\omega t_{n}-\mu_{\cos} & \sin\omega t_{n}-\mu_{\sin} \end{array}\right)$$

a centering of the rhythmic terms, where the intuition behind this is that $Z_{2}$ represents regression residuals, as remarked above. Proceeding with evaluating Equation (3), we have:

$$\beta_{2}^{T}Z_{2}^{T}Z_{2}\beta_{2}=n\beta_{2}^{T}\left(\begin{array}{cc} \sigma_{\cos}^{2} & \sigma_{\cos,\sin}\\ \sigma_{\cos,\sin} & \sigma_{\sin}^{2} \end{array}\right)\beta_{2}$$

where we have defined $\sigma_{\cos}^{2}=\frac{1}{n}\sum_{i}\cos^{2}\omega t_{i}-\mu_{\cos}^{2}$, $\sigma_{\sin}^{2}=\frac{1}{n}\sum_{i}\sin^{2}\omega t_{i}-\mu_{\sin}^{2}$, and $\sigma_{\cos,\sin}=\frac{1}{n}\sum_{i}\cos\omega t_{i}\sin\omega t_{i}-\mu_{\cos}\mu_{\sin}$ as the population (co)variances of the rhythmic terms of the model. Standard algebra for trigonometry and for (population) variance of linear combinations then gives $\beta_{2}^{T}Z_{2}^{T}Z_{2}\beta_{2}={\mathrm{nA}}^{2}\sigma_{\text{cosinor}}^{2},$ where we have defined the cosinor population variance $\sigma_{\text{cosinor}}^{2}=\frac{1}{n}\sum_{i}\cos^{2}\left(\omega t_{i}-\phi \right)-\mu_{\text{cosinor}}^{2}$, with $\mu_{\text{cosinor}}=\frac{1}{n}\sum_{i}\cos\left(\omega t_{i}-\phi \right)$. Then we have the main result:

(4) $$\delta^{2}=\frac{nA^{2}}{\sigma^{2}}\sigma_{\text{cosinor}}^{2}$$

Note that naturally, ${\hat{\beta }}_{2}^{T}Z_{2}^{T}Z_{2}{\hat{\beta }}_{2}=n{\hat{A}}^{2}{\hat{\sigma }}_{\text{cosinor}}^{2}$ also holds for estimated parameters. In the accompanying R script, we give a numerical example that the reduction in sum of squares indeed corresponds to our corrected expression, and not the one given by Zong et al. [1]. Standard cosinor power analysis should thus be based on the noncentral $F\left(2,n-3,\delta^{2}\right)$ distribution. The difference from Lemma 3 and Theorem 2 of Zong et al. [1] is that our Equation (4) contains a population variance instead of a simple sum. We conclude this Commentary by discussing some consequences.

### 2.4 Discrepancy for Non‐Centered Designs

An evenly‐spaced design means that measurements span an integer number of periods, have a constant interval between time points including the interval created when wrapping the end of the measurement span to its beginning, and an equal number of measurements per time point [1]. This design is sufficient (but not necessary!) to ensure that it is both centered, and also that $\sigma_{\cos}^{2}=\sigma_{\sin}^{2}=1/2$ and $\sigma_{\cos,\sin}=0$, so that the noncentrality parameter and consequently experimental power are independent of phase φ: this is termed phase‐invariant design. Because of its centered property, this design yields the same noncentrality parameter as in the article by Zong et al. [1]. The numerical studies that directly validated their analytical expression for experimental power analysis were only based on evenly‐spaced designs, allowing the error to go undetected.

Under controlled laboratory conditions, it is reasonably feasible to realize evely‐spaced designs, although a recent meta‐analysis of time‐resolved RNA‐Seq based analyses of mouse liver gene expression exemplified that non‐evenly‐spaced designs are not necessarily uncommon [3]. Further, as highlighted by Zong et al. [1], under less controlled conditions evenly‐spaced designs can be difficult to realize, or as for the gene expression data from post‐mortem human samples analyzed in their study, entirely infeasible. In such cases the correct noncentrality parameter (Equation 4) is crucial. As an illustration, consider the following *gedankenexperiment*: if the noncentrality parameter in Theorem 2 of Zong et al. [1] were correct, one might place all samples quite densely around the maximum or minimum of the cosine terms in the sum, and expect to achieve maximal power. A moment's reflection will convince the reader that the low curvature around the cosine peak of such a design would make it minimally distinguishable from a fit to a constant term and thus on the contrary result in a low power. Indeed, such a design will result in a low population variance $\sigma_{\text{cosinor}}^{2}$ and Equation (4) thus correctly expresses this low power. A numerical illustration, provided in the accompanying demonstration R script, has 24 timepoint placed uniformly between ZT 5 and 7, assuming a true phase (maximum) at ZT 6. With A/σ=3, this results in a $\delta^{2}$ value of 210 using the expression in Theorem 2 of Zong et al. [1], corresponding to a power virtually 1 for a size (probability of falsely rejecting the null hypothesis) of 0.05 for the null model. However, with the correct expression, Equation (4), we obtain a $\delta^{2}$ value of 0.026, corresponding to a power of 0.052 at size 0.05, which is almost indistinguishable from random chance. This is a contrived example, but it illustrates that the correct form of the noncentrality parameter matters. Real world examples of non‐centered, non‐phase‐invariant designs, such as the human post‐mortem data analyzed by Zong et al. [1], do not generally exhibit such extreme clustering around specific circadian or zeitgeber time points. Still, those analyses should be revisited and corrected using Equation (4).

To summarize, we provide the correct form of the noncentrality parameter necessary for experimental power analysis of the standard cosinor model. In addition, we present more general formulae (Equations (1), (2), (3)) that may serve as a foundation for nonstandard cosinor models that incorporate trends or blocks, say. For nonstandard cosinor models, the general form for the noncentrality parameter (Equation 3) should be used, of which the standard cosinor noncentrality parameter, Equation (4), is a special case. We hope that our discussion stimulates further interest in and development of the cosinor power analysis initiated by Zong et al. [1].

## Conflicts of Interest

The author declares no conflicts of interest.

## Supporting information


**Data S1:** Supporting Information.

## Data Availability

The data that supports the findings of this study are available in the Supporting Information of this article.
